# Evidence for biphasic uncoating during HIV-1 infection from a novel imaging assay

**DOI:** 10.1186/1742-4690-10-70

**Published:** 2013-07-09

**Authors:** Hongzhan Xu, Tamera Franks, Gregory Gibson, Kelly Huber, Nadia Rahm, Caterina Strambio De Castillia, Jeremy Luban, Christopher Aiken, Simon Watkins, Nicolas Sluis-Cremer, Zandrea Ambrose

**Affiliations:** 1 Division of Infectious Diseases, Department of Medicine, University of Pittsburgh School of Medicine, Pittsburgh, PA, 15261, USA; 2 Department of Cell Biology and Molecular Physiology, University of Pittsburgh School of Medicine, Pittsburgh, PA, 15261, USA; 3 Institute for Research in Biomedicine, Bellinzona, CH-6500, Switzerland; 4 Program in Molecular Medicine, University of Massachusetts Medical School, Worcester, MA, 01605, USA; 5 Department of Pathology, Microbiology and Immunology, Vanderbilt University School of Medicine, Nashville, TN, 37232, USA

## Abstract

**Background:**

Uncoating of the HIV-1 core plays a critical role during early post-fusion stages of infection but is poorly understood. Microscopy-based assays are unable to easily distinguish between intact and partially uncoated viral cores.

**Results:**

In this study, we used 5-ethynyl uridine (EU) to label viral-associated RNA during HIV production. At early time points after infection with EU-labeled virions, the viral-associated RNA was stained with an EU-specific dye and was detected by confocal microscopy together with viral proteins. We observed that detection of the viral-associated RNA was specific for EU-labeled virions, was detected only after viral fusion with target cells, and occurred after an initial opening of the core. *In vitro* staining of cores showed that the opening of the core allowed the small molecule dye, but not RNase A or antibodies, inside. Also, staining of the viral-associated RNA, which is co-localized with nucleocapsid, decays over time after viral infection. The decay rate of RNA staining is dependent on capsid (CA) stability, which was altered by CA mutations or a small molecule inducer of HIV-1 uncoating. While the staining of EU-labeled RNA was not affected by inhibition of reverse transcription, the kinetics of core opening of different CA mutants correlated with initiation of reverse transcription. Analysis of the E45A CA mutant suggests that initial core opening is independent of complete capsid disassembly.

**Conclusions:**

Taken together, our results establish a novel RNA accessibility-based assay that detects an early event in HIV-1 uncoating and can be used to further define this process.

## Background

The replication cycle of HIV-1 is complex; while many of the critical steps have been described in great detail, some, including uncoating of the viral core, remain poorly understood. After fusion with the host cell, HIV-1 releases the core into the cytoplasm. The core contains the conical viral capsid, composed of a polymer of capsid protein (CA) subunits, encasing the viral RNA (vRNA) genome. The viral RNA undergoes reverse transcription, forming viral DNA (vDNA) in the cytoplasm. The vDNA together with the nucleocapsid (NC), reverse transcriptase (RT), Vpr, and integrase (IN) form the pre-integration complex (PIC). The PIC is transported to the nucleus by way of microtubules and actin filaments in the cytoplasm [1,2], and subsequently enters the nucleus by mechanisms which have only recently begun to be examined in detail [3]. Inside the nucleus, the HIV-1 DNA is integrated into host cell chromatin, after which the provirus is transcribed for viral protein expression for particle assembly and release from the cell.

The stability of the HIV-1 capsid has been linked to reverse transcription and nuclear entry. CA mutations that alter the intrinsic stability of the capsid have profound effects on reverse transcription [4] and entry of viral DNA into the nucleus [5-8]. In addition, the rhesus macaque tripartite interaction motif 5α protein (rhTRIM5α) is a restriction factor that inhibits HIV-1 and other retroviruses by targeting the viral capsid and inhibiting reverse transcription [9], likely by perturbing the capsid structure. Proteasome inhibitors relieve the block to reverse transcription by rhTRIM5α, suggesting that this restriction targets the reverse transcription complex (RTC) for proteasomal degradation [10]. In addition, the RTC requires cellular factors for completion of reverse transcription that are independent of CA mutations that alter core stability [11,12]. Despite the importance of the structure and stability of the viral capsid in HIV-1 infection, the process of uncoating, which we define as dissociation of the capsid from the core, remains poorly understood, mainly owing to difficulties in the detection of HIV-1 cores soon after entry into target cells. A recent study described the development of assays measuring association of HIV-1 Vpr and CA in HeLa cells, and the timing of escape from TRIMCyp-mediated restriction in owl monkey cells. Using these novel approaches, the authors reported that uncoating could be delayed for a virus with a hyperstable capsid or by preventing reverse transcription, further reinforcing the functional connection between HIV-1 uncoating and reverse transcription [13].

Despite recent advances in studies of the structure and stability of the HIV-1 capsid, uncoating remains poorly understood and is not currently possible to study by live-cell imaging techniques due to lack of available methods to label CA molecules without perturbing the function of the viral capsid. Fluorescence microscopic methods to track the core and viral protein or DNA components of the RTC/PIC have been employed previously [1,2,14-16], but are limited in terms of sensitivity. To circumvent these problems, we applied an alternative method of labeling HIV-1 RNA such that it could be stained with a fluorescent small molecule dye after capsid dissociation *in vitro* or during cell infection. The dye is specific for virus particle-associated RNA and can only access the viral nucleic acid after an initial uncoating step that appears to involve opening of the capsid. We observed that the kinetics of RNA staining were altered by the presence of rhTRIM5α or TRIMCyp, by CA substitutions altering capsid stability, and by addition of a small molecule inducer of HIV-1 uncoating. Surprisingly, the previously identified E45A CA mutant, which exhibits hyperstable cores *in vitro*, exhibited efficient staining of viral-associated RNA through cores *in vitro* and accelerated staining kinetics *in vivo*, suggestive of a permeable capsid.

## Results

### HIV-1 RNA staining in virions requires CA core opening

To label and track HIV-1 RNA intracellularly, we incorporated a modified nucleoside, 5-ethynyl uridine (EU), into vRNA during virus production. Detection of alkyne-modified nucleosides in vRNA was performed by selective ligation of an azide-containing fluorescent dye to the modified nucleoside, in a so-called a “click” reaction [17], and subsequent fluorescent confocal microscopy of infected target cells. Because HIV-1 particles can incorporate cellular mRNA from the producer cell [18-20], the modified virus-associated RNA could, in principle, be both cellular and viral in origin.

HIV-1 particles were made by transfecting 293T cells with a proviral construct and a plasmid encoding VSV-G in the presence of EU for different lengths of time to optimize vRNA labeling and normal viral protein production. EU was added to the transfection medium for the first 16 h of transfection, the last 24 h of transfection, or the entire 48 h of transfection. To test whether EU-labeled viruses were infectious, viruses produced in the presence of EU incorporation for different lengths of time were assayed for infectivity in HIV-1 indicator cells as well as for total CA production. For HIV-1 produced in the presence of 1 mM EU for 16 h, the specific infectivity, or infectious units (IU) per ng of CA antigen, was comparable to that of control virus made in the absence of EU (Table 1). Viruses produced in the presence of EU during the entire 48 h of transfection or for the last 24 h of transfection exhibited 2- and 3-fold lower infectivity relative to unlabeled HIV-1. Furthermore, decreased yields of virus were produced during extended culture time in EU, which was likely due to increased cellular toxicity of 293T cells incubated for longer periods of time with 1 mM EU (Additional file 1: Figure S1). Subsequent experiments were performed using 0.1 - 0.4 mM EU during 48 h of virus production to minimize cell toxicity. Viral DNA from cells infected with EU-labeled virus was sequenced and revealed no difference in mutations compared to unlabeled virus (data not shown), suggesting that EU incorporation does not affect reverse transcription.

**Table 1:** Specific infectivity (infectious units per ng p24) of EU-labeled viruses

**Virus**	**p24 (ng/ml) ± SD**	**Specific infectivity**
No DNA	<0.008	0
No EU	191 ± 3	1510
EU for 1^st^ 16 h	28 ± 4	2230
EU for 2^nd^ 24 h	165 ± 4	500
EU for 48 h	20 ± 1	820
D443N	107 ± 10	8

SD, standard deviation of duplicate measurements.

**Figure 1:**
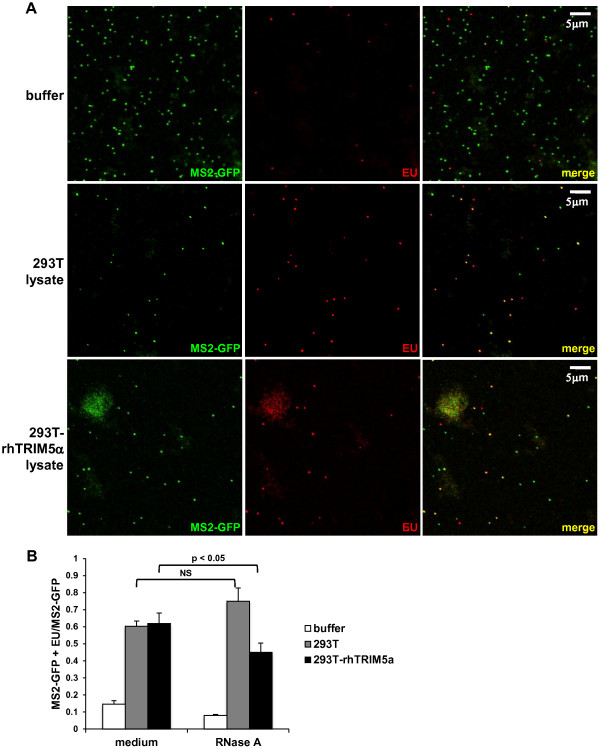
**EU staining of HIV-1 RNA *in vitro*. (A)** EU-labeled virus encoding MS2-binding sites and produced in the presence of MS2-GFP (green) was treated with cell extraction buffer, 293T cell lysate, or 293T cell lysate containing rhTRIM5α for 15 minutes, fixed onto slides, and then stained for EU (red). Particles were visualized by fluorescent confocal microscopy and shown for each individual color and with both colors (merge). **(B)** MS2-GFP+ and EU+ virions were counted per field after treatment with buffer, 293T cell extract, or 293T cell extract expressing rhTRIM5α in the presence of 10 μg/ml RNase A. Data represent the ratio of double positive particles of the total counted MS2-GFP+ particles ± SEM of at least 4 fields. Results are representative of 2 independent experiments. A student’s t test was performed on medium vs. RNase A treatment of virions incubated with either 293T or 293T-rhTRIM5α lysate. NS denotes a non-significant p value (>0.05).

To determine if the EU-binding dye could penetrate intact cores, we treated EU-labeled HIV-1 particles with cell extraction buffer or buffer containing lysates from 293T cells or 293T cells expressing rhTRIM5α. The restriction factor rhTRIM5α has been reported to disrupt HIV-1 cores and assembled CA tubes *in vitro*[21-23]. To visualize stained and unstained particles, infectious virus containing a genome with MS2 protein binding sites was produced in cells expressing the MS2-GFP fusion protein and incubated with EU. Co-localization of vRNA staining and MS2-GFP was greater than 75%, whereas particles without MS2-binding sites showed a background of 8% co-localization of EU staining and GFP (Additional file 1: Table S1 and Figure S2). Particles were fixed to slides and stained for EU (Figure 1A). Few particles stained for vRNA when treated with buffer alone, which permeabilizes the viral membrane. However, normal cell lysate increased staining of the vRNA, suggesting that cellular factors may promote dye entry into the core. vRNA staining was also observed in particles subjected to cell lysate containing rhTRIM5α, which leads to disruption of the capsid. Without an intact CA core to protect it, vRNA should be sensitive to RNase A treatment, leading to its degradation. With RNase A added to the particles exposed to rhTRIM5α, significantly fewer vRNA puncta were detected by staining *in vitro* compared to virions without RNase A treatment (Figure 1B). However, virions treated with normal 293T cell lysate and RNase A showed no decrease in vRNA staining, suggesting that the small dye (approximately 268 Da), but not the larger enzyme (12.7 kDa), could access the RNA. Particles made without MS2-GFP also showed significant loss of vRNA staining in the presence of 10 and 100 μg/ml RNase A (Additional file 1: Figure S3).

### Labeled HIV-1 RNA is detected in infected cells

**Figure 2:**
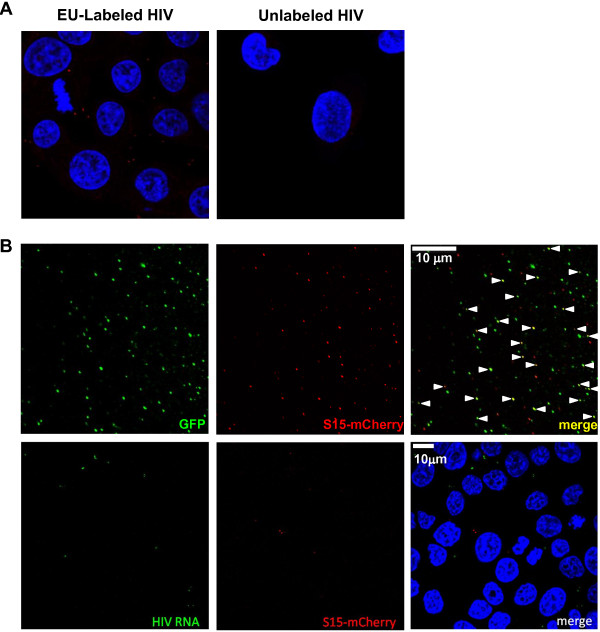
**EU-labeled HIV-1 can be visualized intracellularly. (A)** RNA staining (red) of TZM-bl cells after infection for 2 h with equal multiplicities of infection of EU-labeled virus (labeled during the first 16 h of transfection) or unlabeled virus. **(B)** Viral membrane-associated S15-mCherry (red) is incorporated into MS2-GFP+ (green) virions prior to infection (top), but is not co-localized with EU-labeled WT HIV-1 RNA staining (green) after incubation with cells (bottom). Viruses labeled with EU and S15-mCherry were used to infect TZM-bl cells for 1 h and stained for EU. DAPI was used to stain nuclei (blue). MS2-GFP or EU and S15-mCherry are shown individually and overlayed together (merge). The panels are representative of multiple fields.

To visualize EU-labeled virus-associated RNA in cells, TZM-bl cells were infected with virus-containing transfection supernatants and fixed, permeabilized, and stained. Viruses produced in the presence or absence of EU were incubated with TZM-bl cells for 2 h before washing and staining (Figure 2A). The cells infected with EU-labeled virus had detectable punctate staining in the cytoplasm, whereas the cells infected with unlabeled virus did not. Uninfected cells did not show any staining (data not shown). Pseudotyping HIV-1 with the vesicular stomatis virus glycoprotein G (VSV-G) was used to ensure high infectivity levels of the virus and to compare different cell lines that may not have significant levels of CD4 or co-receptors. VSV-G allows viral entry via endocytosis rather than cell membrane fusion by HIV-1 envelope [24,25]. Fusion rates assessed by imaging studies have shown VSV-G to be accelerated compared to HIV-1 CXCR4-tropic envelope (15 minutes vs. 48 minutes) [13]. While it is unknown whether the route of entry will affect downstream events, the previous study did not show a difference in the timing of intracellular uncoating based on entry pathway.

To determine whether or not virus-associated RNA staining detected HIV-1 prior to fusion and entry into cells, virus was produced in the presence of EU and the membrane-associated S15-Cherry protein [26]. Thus, vRNA was labeled with EU (green) and viral membranes were labeled with S15-mCherry (red). A loss of mCherry signal indicates that virus particles have fused with the cell or endosomal membrane. S15-mCherry was efficiently incorporated into virions as shown in particles containing a packagable viral genome with binding sites for the bacteriophage MS2 protein and produced in cells expressing MS2-GFP fusion protein (Figure 2B). When EU- and mCherry-labeled particles were added to cells, some mCherry particles on or between cells were observed even after extensive washing. However, vRNA staining was not co-localized with mCherry in any fields, suggesting that the EU staining was specific for intracellular virus-associated RNA. This was expected, as the dye does not permeate through lipid bilayers, including viral membranes.

### Labeled HIV-1 RNA maintains co-localization with NC but not Vpr or CA after infection

**Figure 3:**
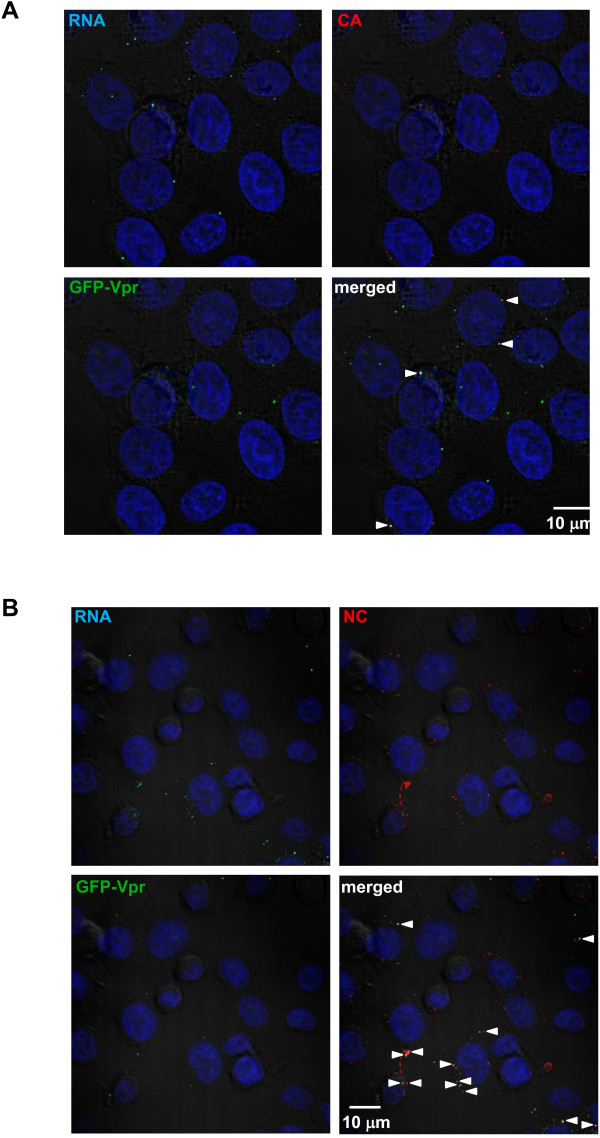
**EU-labeled WT HIV-1 RNA is co-localized with NC in cells.** TZM-bl cells were infected for 1 h with EU-labeled virus containing GFP-Vpr (green) and stained for **(A)** RNA (cyan) and CA (red) or **(B)** RNA (cyan) and NC (red). DAPI was used to stain nuclei (blue). Cells were then visualized by fluorescent confocal microscopy. Arrows indicate co-localization of vRNA with CA or NC staining when both colors are merged (white). Results are representative of multiple independent experiments.

**Figure 4:**
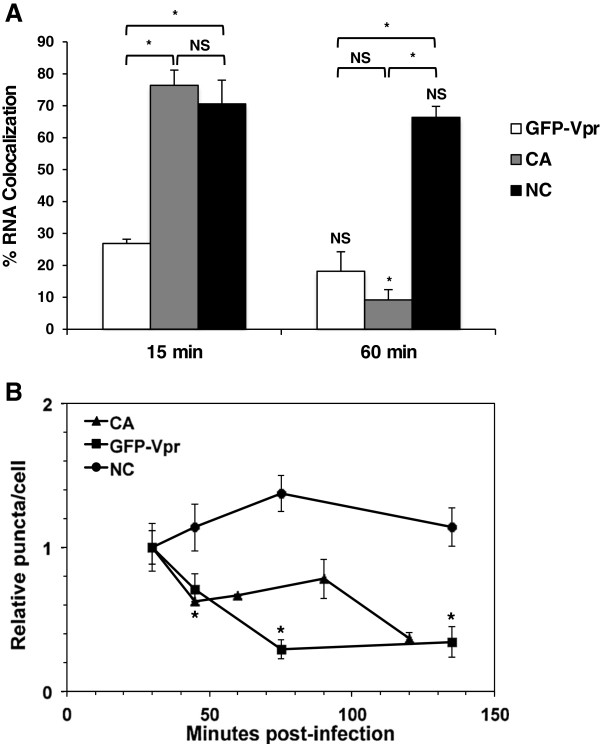
**Quantification of viral proteins co-localized with WT HIV-1 RNA over time in cells. (A)** After HIV-1 infection of TZM-bl cells, RNA staining was counted alone and when co-localized with CA, GFP-Vpr, or NC at 15 min and 60 min post-infection. The percentage of co-localized events for at least four fields was calculated. **(B)** CA, GFP-Vpr, and NC puncta were quantified at multiple time points post-infection. The first time point was normalized to 1. Data represent the mean ± SEM of at least 4 fields. Asterisks denote statistically significant p values (p < 0.05) between time points for each viral protein or at each time point, comparing NC with CA or with GFP-Vpr. NS denotes a non-significant p value (>0.05). Results are representative of 2 independent experiments.

Analysis of GFP-Vpr-labeled HIV-1 virions detected GFP-Vpr in association with CA in approximately 80% of particles [2]. Following HIV-1 entry into the cytoplasm, CA and GFP-Vpr co-localization decreases over time, which is thought to be due to uncoating [13]. Therefore, we asked whether EU-labeled vRNA of incoming particles would be co-localized with CA and GFP-Vpr. Cells were inoculated with EU-labeled virus that also contained co-packaged GFP-Vpr, and were subsequently stained for EU and with antibodies against CA (Figure 3A). Many particles exhibited RNA staining, CA staining, or GFP-Vpr alone. At 15 minutes post-infection, the percentage of EU staining that was co-localized with CA or with GFP-Vpr was 76% or 27%, respectively (Figure 4A). However, at 1 hour post-infection the percentage of EU staining with CA decreased significantly to 9% and decreased slightly with GFP-Vpr to 18% of the time. This is consistent with the hypothesis that CA molecules are rapidly removed from the core during uncoating. The frequency of detectable CA and GFP-Vpr puncta declined over time (Figure 4B), suggesting that cores had uncoated and these RTC components had diffused in the cytosol. Accordingly, we observed diffuse signals for EU-labeled RNA at later time points after infection as compared to mostly bright, punctate staining at earlier time points (Additional file 1: Figure S4).

HIV-1 nucleocapsid (NC) has been shown to bind vRNA and act as a chaperone during vDNA synthesis [27]. Therefore, vRNA should remain associated with NC in the core and throughout reverse transcription after uncoating. To determine if EU-labeled vRNA was co-localized with NC, staining for NC by monoclonal antibodies together with EU staining was performed in HIV-1 infected cells (Figure 3B). While NC and GFP-Vpr could be detected alone, vRNA staining was co-localized with NC much more than with GFP-Vpr or CA at 1 h post-infection. NC and vRNA co-localization occurred 71% after 15 minutes and 66% after 1 h post-infection (Figure 4A), suggesting that EU detection was specific for virus-associated RNA. By contrast to GFP-Vpr and CA, NC detection did not diminish over time in the infected cells, suggesting that the particles were not being totally degraded (Figure 4B).

### Intracellular HIV-1 RNA staining increases within 30 minutes post-infection and is affected by rhTRIM5α and TRIMCyp

**Figure 5:**
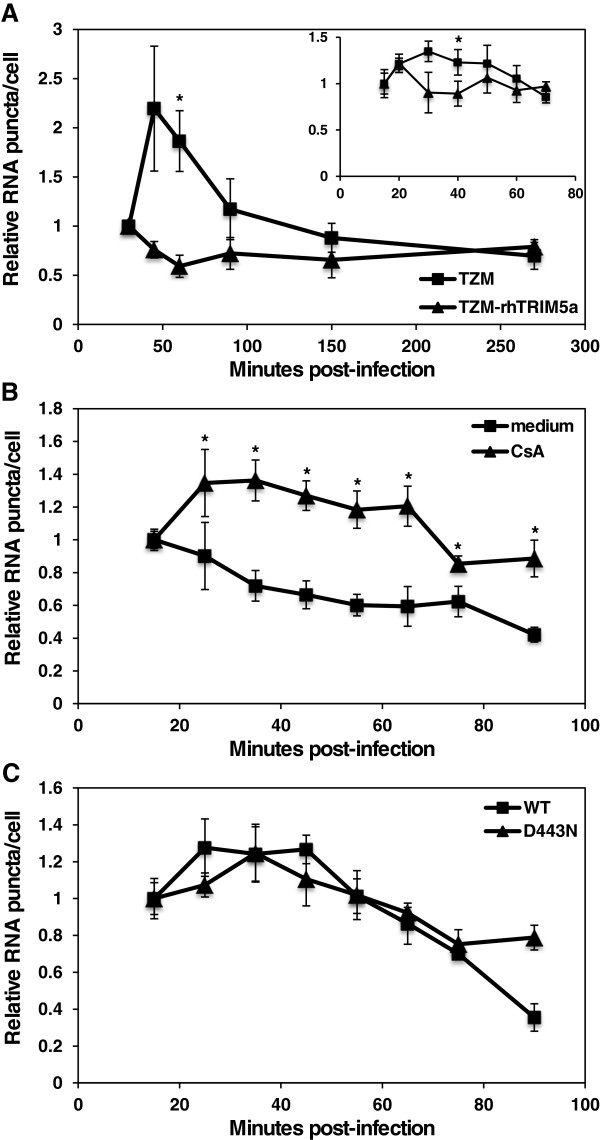
**The kinetics of HIV-1 RNA staining is affected by rhTRIM5α/TRIMCyp but independent of reverse transcription. (A)** TZM-bl cells or TZM-bl cells expressing rhTRIM5α were infected with WT HIV-1 and stained for EU at multiple time points. The inset shows staining from a second experiment at earlier time points. **(B)** OMK cells were infected with WT HIV-1 in the presence or absence of CsA and stained for EU at multiple time points. **(C)** TZM-bl cells were infected with WT HIV-1 or the D443N RT mutant and stained for EU at multiple time points. 0.6 - 13.5 vRNA puncta were detected per cell. Data represent the mean ± SEM of at least 4 fields and the first time point was normalized to 1. Asterisks denote statistically significant p values (p < 0.05) at each time point between the two lines. The graphs are representative of 2 independent experiments.

Next we performed a time course in which TZM-bl cells were infected with WT HIV-1 in a synchronized manner for 15–30 minutes and fixed at different time points thereafter. Virus-associated RNA was stained and quantified (Figure 5A). The number of counted EU-stained spots per cell, as measured by DAPI staining of nuclei, was determined for 4 fields for each time point with approximately 10 – 40 cells counted per field. We observed an average of 0.6 – 10 EU-stained spots per cell after 15 – 30 minutes of incubation with the various viruses and conditions tested (Additional file 1: Figure S5). The number of vRNA puncta decreased between 45 and 150 minutes post-infection, which was coincident with diffusion of the signal over time (Additional file 1: Figure S4). The kinetics of vRNA detection after HIV-1 infection of TZM-bl cells showed a reproducible initial increase in EU-containing vRNA staining from 30 to 45 minutes, followed by a decrease thereafter. Because new vRNA molecules likely cannot be synthesized during early post-entry time points, it is possible that the AlexaFluor azide stain requires opening of the CA core in the cytoplasm in order to gain access to the viral genome, consistent with the results of staining viruses *in vitro* (Figure 1). A similar effect is seen for antibody staining of NC (Figure 4B), which is presumably bound to vRNA inside the core. An increase in GFP-Vpr or CA staining was not observed.

rhTRIM5α has been shown to bind to HIV-1 capsids and to restrict HIV-1 infection prior to reverse transcription *in vivo*[9,28]. This restriction factor has also been reported to disrupt HIV-1 cores and assembled CA tubes *in vitro*[21-23]. We therefore examined the effects of rhTRIM5α on the kinetics of staining of virus-associated RNA. When cells expressing rhTRIM5α were infected with EU-labeled viruses, we observed a small initial increase in the number of detectable vRNA between 15 – 25 min post-infection that decreased rapidly, and the low level of staining remained fairly stable during the time course of up to 270 minutes (Figure 5A). This difference compared to normal TZM-bl cells may be due to more rapid and complete disruption of the incoming CA cores by the restriction factor, allowing the stain to access vRNA earlier.

Similarly, owl monkey kidney (OMK) cells express TRIMCyp, a fusion protein of TRIM5α and cyclophilin A, that restricts HIV-1 infection and can be reversed by inhibiting TRIMCyp binding to CA with the drug cyclosporine A (CsA) [29,30]. OMK cells were infected with EU-labeled WT HIV-1 and stained for RNA between 15 – 90 minutes post-infection (Figure 5B). Like cells expressing rhTRIM5α, these cells expressing TRIMCyp did not show an early increase in RNA staining. This staining pattern was reversed in cells treated with CsA, suggesting that when the TRIMCyp restriction was relieved, there was a delay in vRNA staining owing to restoration of normal uncoating.

The RNase H activity of RT cleaves the vRNA in RNA-DNA hybrids formed during reverse transcription, and we wondered whether RNase H-dependent degradation is responsible for the observed decrease in vRNA staining over time. To test this, we made EU-labeled HIV-1 containing the D443N amino acid substitution in RT. This mutant leads to a loss of RNase H activity, leading to incomplete reverse transcription [31]. As a result, this mutant had almost a 190-fold decrease in specific infectivity as compared to WT HIV-1 (Table 1), despite having similar levels of viral particles. The vRNA staining assay revealed that D443N HIV-1 had a similar decay curve as WT HIV-1 in TZM-bl cells (Figure 5C), indicating that vRNA degradation was not due to viral RNase H activity. These results also indicated that premature termination of reverse transcription does not alter the kinetics of vRNA staining.

### Perturbation of HIV-1 CA capsid stability by mutations or a drug alters vRNA staining kinetics

**Figure 6:**
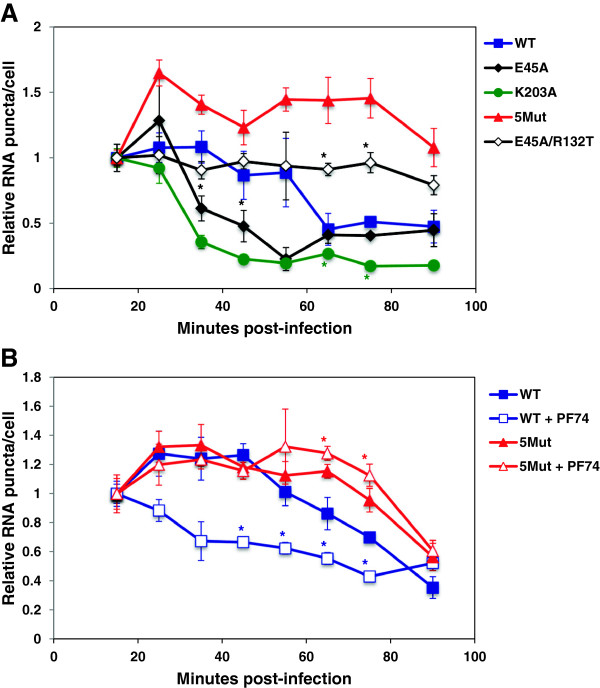
**Viral RNA staining is altered by mutations that affect capsid stability.** TZM-bl cells were infected with **(A)** EU-labeled WT, E45A, K203A, 5Mut, and E45A/R132T HIV-1; or **(B)** EU-labeled WT and 5Mut HIV-1 in the presence and absence of 10 μM PF74. 0.6 - 4.4 vRNA puncta were detected per cell. Data represent the mean ± SEM of at least 4 fields and the first time point was normalized to 1. Asterisks denote statistically significant p values (p < 0.05) between WT and each mutant or with or without PF74 treatment at each time point. The graphs are representative of 2 independent experiments.

To determine whether vRNA staining kinetics reflect core opening after HIV-1 infection, we stained vRNA at multiple time points after infection of TZM-bl cells with HIV-1 CA mutants exhibiting alterations in capsid stability (Figure 6A). The CA K203A mutant was previously shown to have highly unstable cores and low infectivity [4]. K203A vRNA staining began to decay after 25 minutes post-infection instead of after 55 minutes post-infection observed for WT HIV-1. Moreover, K203A HIV-1 did not show the usual transient increase in vRNA staining typically observed for wild type HIV-1, suggesting that CA core opening for this mutant occurred within 15 – 25 minutes post-infection. This observation was similar to the data for WT HIV-1 in rhTRIM5α-expressing cells (Figure 5A). A quintuple mutant, Q67H/K70R/H87P/T107N/L111I, designated 5Mut and recently described as having a hyperstable capsid [32], showed stable vRNA for up to 75 minutes post-infection (Figure 6A). Together, these data suggest that the kinetics of vRNA staining and decay are related to HIV-1 uncoating during infection.

Interestingly, E45A HIV-1, which had previously been described as having a hyperstable capsid and lower infectivity as compared to WT HIV-1 [4,33], exhibited a rapid vRNA decay profile that was similar to that of K203A. However, unlike the K203A mutant, E45A HIV-1 had an increase in vRNA staining between 15 – 25 minutes post-infection, followed by a significant decline at 35 minutes post-infection (Figure 6A). These data indicate that the capsid of E45A HIV-1 dissociated early after infection. The E45A phenotype was partially reversed with the addition of the mutation of R132T, resulting in a double mutant for which EU staining resembled WT HIV-1. The E45A/R132T mutant is more infectious than the E45A mutant, indicating that the functional defect induced by E45A is partially rescued by R132T [34].

To further examine whether capsid stability affects the kinetics of vRNA decay, we tested the effect of PF74, a small molecule inhibitor that destabilizes the HIV-1 capsid [32,35], in our assay (Figure 6B). We infected TZM-bl cells with WT HIV-1 in the presence of PF74 and observed accelerated vRNA decay relative to infection in the absence of the compound. Whereas WT vRNA staining began to decay after 45 minutes post-infection in untreated cells, PF74 led to lower vRNA staining at the first time point, showing no transient increase in vRNA staining that is observed with most viruses in cells without the CA inhibitor, similar to the unstable K203A mutant. In contrast, the PF74-resistant 5Mut virus [32] was efficiently stained with the EU dye for extended times, and its staining was unaffected by infection in the presence of PF74.

### CA E45A HIV-1 cores are more permeable than WT HIV-1 cores

**Figure 7:**
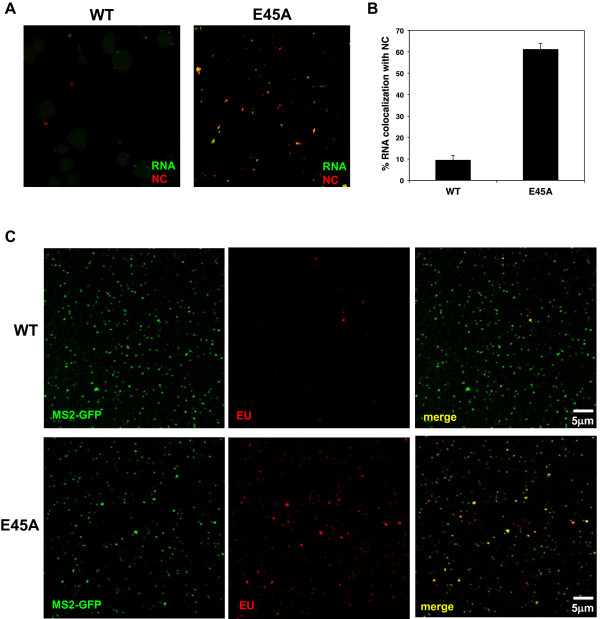
**E45A HIV-1 cores allow vRNA and NC staining more readily than WT HIV-1 cores. (A)** Equal amounts of p24 of EU-labeled WT HIV-1 and E45A HIV-1 were treated with cell extraction buffer for 15 minutes, fixed onto slides, and then stained for EU (green) and NC (red). Particles were visualized by fluorescent confocal microscopy. **(B)** RNA staining of WT HIV-1 and E45A HIV-1 was counted alone and when co-localized with NC. The percentage of co-localized events for at least four fields was calculated. Data represent the mean ± SEM of at least 4 fields. **(C)** Equal amounts of p24 of EU-labeled WT HIV-1 and E45A HIV-1 also containing MS2-GFP (green) were treated with cell extraction buffer for 15 minutes, fixed, and stained for EU (red). Particles were visualized by fluorescent confocal microscopy and shown for each individual color and with both colors (merge).

Because of the unexpected rapid RNA staining of E45A HIV-1, we hypothesized that the integrity of E45A capsid is compromised by the mutation, allowing molecules, such as antibodies and the fluorescent dye, to enter the core more rapidly. To determine whether E45A cores allow entry of different molecules into the core more easily than WT CA cores, equal amounts of EU-labeled WT HIV-1 and E45A HIV-1 particles in buffer (2.5 ng CA) were fixed onto slides and stained for EU by the small dye and for NC by monoclonal antibodies (Figure 7A). Similar to the previous results shown in Figure 2A, we observed little RNA staining of WT vRNA. In addition, we saw almost no staining of NC for the WT virions. However, we observed both viral-associated RNA and NC staining in E45A virions, suggesting that both the small molecule dye and the anti-NC antibodies could penetrate the cores. The co-localization of NC and viral-associated RNA in E45A cores was approximately 65%, whereas it was only approximately 10% for WT cores (Figure 7B). In addition, EU-labeled E45A HIV-1 particles containing MS2-GFP were stained and showed higher levels of staining compared to WT MS2-GFP particles (Figure 7C).

**Figure 8:**
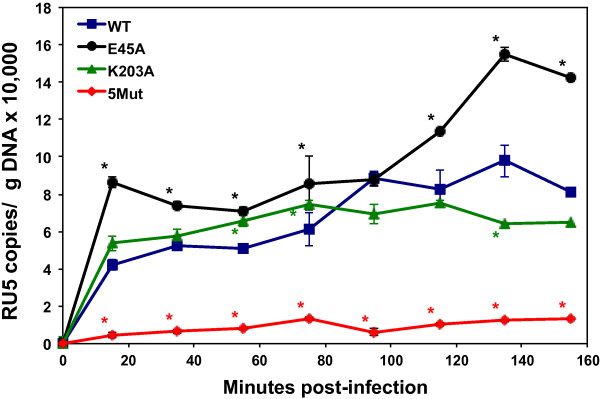
**Initiation of reverse transcription was more rapid in E45A HIV-1 compared to WT, K203A, and 5Mut HIV-1.** Early viral reverse transcripts (RU5) were measured by qPCR from TZM-bl cells infected with equal p24 levels of WT, E45A, K203A, and 5Mut HIV-1. Data represent the mean ± SEM of duplicates and are normalized for μg of total isolated DNA. Asterisks denote statistically significant p values (p < 0.05) between WT and each mutant at each time point. The graph is representative of 2 independent experiments.

Because HIV-1 requires a cell factor(s) for completion of reverse transcription [12], it is possible that premature opening of the core might lead to earlier initiation of reverse transcription. If this is the case, a core that remains closed longer should exhibit slower production of viral DNA. To test this, we quantified early (RU5) reverse transcription products in cells infected with equal quantities of WT HIV-1 and mutant virions (Figure 8). Indeed, we observed a more rapid accumulation of early reverse transcripts for E45A HIV-1 as compared to WT HIV-1, K203A HIV-1, or 5Mut HIV-1. The level of E45A reverse transcripts was 2-fold higher than the WT vDNA level at 15 minutes post-infection that continued to increase above WT HIV-1 reverse transcripts during the time course. By contrast, the level of reverse transcripts for the K203A and 5Mut viruses did not increase over the 155-minute time course. The results for early reverse transcripts for the K203A mutant were consistent with previous reports looking at significantly later time points [4,5], which may be due to a completely unstable core that leads to poor infectivity for this virus [4,36]. In contrast, E45A HIV-1 has detectable infectivity (20-30-fold lower than WT HIV-1) [6,36], perhaps allowing more efficient initiation of reverse transcription to occur if the core partially uncoated or opened earlier. Late reverse transcripts did not increase significantly for the viruses during this short time course (data not shown).

## Discussion

One aspect of HIV-1 biology that is just beginning to be understood is the process of uncoating of the core following viral entry into the cell. Uncoating appears intimately linked to reverse transcription, as mutations that destabilize the capsid generally impair reverse transcription in target cells [4], as do restrictive TRIM5 proteins [9,37]. Completion of reverse transcription may also require capsid dissociation, allowing the RTC to come into contact with putative positive-acting intracellular factors that facilitate viral DNA synthesis [11,12]. Reverse transcription may also promote later stages of uncoating; perhaps the size and rigidity of double-stranded viral DNA formed during reverse transcription may help to destabilize the CA lattice and may not physically fit inside of the core [13]. However, a structural and molecular understanding of uncoating and its role in reverse transcription remain unresolved.

In the present study, we describe the detection of intracellular HIV-associated RNA during infection. The modified nucleoside, EU, is incorporated into viral and cellular RNA in the producer cell. Fluorescent azide molecules can efficiently and selectively engage RNA-incorporated EU in a “click” reaction after entry into the CA core. We did not observe significant background staining of uninfected cells or cells infected with unlabeled HIV-1. Neither the virus-associated RNA signal nor the decay of this signal required reverse transcription or viral RNase H activity, which may be the result of a loss of signal of the labeled RNA after dissociation of the CA core and diffusion in the cytosol. The EU signal persisted for up to 4 hours, but the staining became more diffuse over time. vRNA was largely associated with NC but less so with CA or GFP-Vpr, especially as the infection progressed. This was not surprising, since HIV-1 NC favors binding to single-stranded RNA and assists RT with various steps during reverse transcription [27,38]. In contrast, CA has been shown previously to dissociate within 40–60 minutes post-infection, and over 50% of the GFP-Vpr signal is lost between 1 and 2 hours post-infection [13], which is consistent with our results.

Our assay showed a steady, reproducible loss of HIV-1 RNA detection with time, consistent with uncoating and the proposed instability of HIV-1 RNA in the cytosol [39]. The kinetics were affected by manipulating capsid stability by mutations or a small molecule CA inhibitor. The K203A CA mutant, which has been described as having a more fragile core than WT [4,13], showed more rapid loss of vRNA staining. Similarly, cells infected with WT HIV-1 that were treated with PF74, a capsid-destabilizing compound [32], showed much more rapid vRNA decay than in untreated cells. Conversely, the 5Mut CA mutant that has a very stable core [32] had more persistent detection of EU-labeled RNA that did not decline appreciably for up to 75 minutes post-infection and did not show vDNA accumulation during that time period. 5Mut particles are resistant to PF74 antiviral action and capsid destabilization, which explains the similar vRNA staining that we observed for this virus in the presence and absence of the drug. Interestingly, the infectivity of 5Mut particles is similar to WT [40], suggesting that a hyperstable capsid is not an impediment to HIV-1 infection, at least in some target cells.

While it seems likely that the decay of HIV-1 CA staining in HeLa cells over time also reflects uncoating, as shown recently [13], its rapid decline and loss of signal (t_1/2_ of approximately 1–2 hours post-infection) suggests that the immunochemical assay is detecting a major uncoating event in which most CA molecules disassociate from the RTC within 1 hour post-infection. Due to the difficulty in obtaining reproducible staining quantitation of sufficient number of particles, the CA staining assay lacks the requisite sensitivity to identify subtle changes in capsid structure and the extent of uncoating. By contrast, the EU staining assay relies on RNA accessibility to a small molecule dye, and the rapid increase in staining observed for wild type HIV-1 suggests that RNA cannot be accessed by the dye until an initial event occurs in the presence of cellular factors. This was evident when looking at infection of cells between 15 – 45 minutes post-infection with WT HIV-1 and different CA mutants, and in biochemical studies with permeabilized virions. We also observed an increase in detection of NC, which should be contained within the core, between 30 and 75 minutes after infection. This initial opening did not lead to an immediate loss of CA staining, suggesting that EU staining reflects a more subtle uncoating step occurring prior to bulk dissociation of CA from the core. We also detected cellular accumulation of vDNA only after 75 minutes of virus inoculation, suggesting that reverse transcription proceeds after access to the cytosol.

The putative early uncoating event reflected by RNA staining also appeared to be inhibited by rhTRIM5α and TRIMCyp restriction factors. rhTRIM5α engages the capsid prior to reverse transcription and binds to assembled capsids, leading to their disruption [9,21,23,28]. Similarly, TRIMCyp leads to capsid disassembly that is prevented by CsA treatment [29,30]. Our RNA accessibility assay results are consistent with the interpretation that these restriction factors disrupt the HIV-1 capsid and the effects of TRIMCyp restriction on vRNA staining could be reversed by CsA treatment, which prevents restriction.

Previously, Hulme et al. showed that complete uncoating, as measured by GFP-Vpr and CA co-localization, or by escape from TRIMCyp by timed CsA withdrawal [13], is delayed by treatment by an RT inhibitor, suggesting that reverse transcription promotes uncoating. Our results showed that inhibition of reverse transcription did not alter the kinetics of RNA accessibility, suggesting that the early uncoating event required for RNA exposure is not dependent on reverse transcription and consistent with a recent study showing the fate of viral core components during premature uncoating [39]. It is possible that human cell factors induce an initial uncoating event to allow reverse transcription to continue such that complete dissociation of the core can occur. It is also possible that the early uncoating event results in permeabilization of the core to small molecules, such as dNTPs, required for reverse transcription.

It was surprising that the E45A mutant, which was described as having a more stable capsid than WT HIV-1 [4], consistently exhibited RNA accessibility kinetics that resembled those of mutants with unstable capsids. In contrast to the K203A mutant, cytoplasmic E45A cores initially exhibited a small transient increase in RNA staining between 15 and 25 minutes post-infection, similar to WT cores. This was followed by a rapid decrease in vRNA staining as observed for the unstable K203A mutant. Furthermore, reverse transcription was initiated more rapidly for E45A HIV-1 as compared to WT HIV-1 or the other mutants tested. Importantly, the second-site suppressor mutation, R132T, partially restored the E45A RNA staining kinetics to wild type, correlating the effect of E45A mutation on RNA accessibility with its impairment of infectivity. Our previous results using correlative live-cell microscopy and cryo-electron tomography of infected cells suggested that E45A CA cores disassociate more slowly intracellularly than WT cores [33]. While the comparable behavior of the K203A and E45A mutants appears contradictory, it supports the hypothesis that a subtle HIV-1 CA uncoating event precedes and is independent of a more extensive dissociation of CA from the core. We suggest that for the E45A mutant virus, the first uncoating step occurs more rapidly than for WT HIV-1 but that the second uncoating step is delayed relative to WT HIV-1. The first step would allow an opening for some cellular factors to access the interior of the core, leading to more rapid synthesis of early reverse transcripts, consistent with the results shown here. However, the majority of the E45A CA remains intact, delaying completion of reverse transcription as compared to WT HIV-1. It is possible that a multi-step uncoating process is necessary for both HIV-1 to complete reverse transcription with cellular factors and allowing the RTC to be somewhat contained to prevent disassociation of key components from the complex. Consistently biphasic uncoating of purified HIV-1 cores has been observed biochemically [4]. Alternatively, the lattice of E45A CA cores may be more leaky, allowing small molecules to gain access inside. Further high-resolution studies comparing WT and E45A CA cores are needed to determine if they differ in lattice structure or flexibility.

## Conclusions

In conclusion, we have developed a fluorescence-based RNA accessibility assay that detects an initial opening of the HIV-1 capsid. This initial uncoating step may be triggered by host cell factors and can be influenced by alteration of the stability of the core by CA mutations or a small molecule inhibitor. The RNA accessibility assay, in combination with complementary approaches to detect capsid dissociation, will be useful for studying the role of cellular factors in the early postentry stages of HIV-1 infection.

## Methods

### Cell lines and antiviral compounds

293T cells were used for transfections and for cell lysates described in assays below. TZM-bl cells are HeLa cells expressing CD4, CXCR4, and CCR5 that carry the lacZ and firefly luciferase genes downstream from the HIV-1 LTR [41]. OMK cells are owl monkey kidney cells that have been shown to express endogenous TRIMCyp [29,30]. TZM-rhTRIM5α and 293T-rhTRIM5α cells express rhTRIM5α from the pLPCX-TRIM5a-HA vector, obtained through the NIH AIDS Research and Reference Reagent Program from Drs. Joseph Sodroski and Matt Stremlau. GHOST cells are human osteosarcoma cells expressing CD4, CXCR4, and CCR5 that carry the GFP gene downstream from the HIV-2 LTR [42]. All cells were grown in DMEM and supplemented with 10% fetal bovine serum and 1% penicillin, streptomycin, and L-glutamine. PF-3450074 (PF74) [35] was synthesized by the Vanderbilt Institute of Chemical Biology Synthesis Core, Vanderbilt University, Nashville, TN. PF74 was dissolved as a 10 mM stock in DMSO and added to target cell cultures at a final concentration of 10 μM.

### Virus stocks and EU-incorporation

HIV-1 particles were made by transfection of 293T cells with the following plasmids: pNLdVdE-luc, a plasmid containing the HIV-1_NL4-3_ provirus with an early stop codon in *vpr*, a deletion in *env*, and the firefly luciferase gene in place of *nef*[6]; GFP-Vpr plasmid, a kind gift from Dr. Tom Hope [2]; and pL-VSV-G, a plasmid encoding the envelope glycoprotein from vesicular stomatitis virus (VSV) [43]. Particles were also produced with the S15-mCherry plasmid, a kind gift from Dr. Tom Hope [26]. The pNLdVdE-luc plasmid had the following CA amino acid substitutions introduced by via cloning from other HIV-1_NL4-3_ vectors or PCR mutagenesis: E45A, E45A/R132T, K203A, or 5Mut. Similarly, PCR mutagenesis was used to create pNLdVdE-luc containing the RT mutation D443N. Transfections were performed using calcium phosphate or Lipofectamine 2000 in medium containing 0.1 – 1 mM EU (Invitrogen, Carlsbad, CA) for 48 h. Cell supernatants were collected, ultracentrifuged through a 20% sucrose cushion to remove free EU, resuspended in fresh medium, and frozen at −80°C in aliquots.

Viruses from transfection supernatants were titered in GHOST cells in duplicate. Medium was replaced after 2 h and cells were analyzed after 48 h on an LSRII flow cytometer for GFP expression in <40% of the cells. CA levels were measured by serial dilutions of supernatants by p24 ELISA (Zeptometrix, Buffalo, NY) in duplicate.

Additional experiments showing EU co-localization with virus containing bacteriophage MS2 coat protein binding sites were performed, using a plasmid encoding a viral vector sequence containing twelve MS2-binding sites. The vector was cotransfected with either WT or E45A pcHelpΔVif [44], a kind gift from Dr. Klaus Strebel, pL-VSV-G, and pcDNA-MS2GFP, a vector expressing MS2-GFP, in the presence of 0.4 mM EU. The transfection medium was replaced with 0.1 mM EU an additional 32 h. The EU-labeled virions were harvested by filtering through 0.45 μm syringe filters and purified through a sucrose cushion. The pelleted virions were resuspended in 200 μl of fresh DMEM medium with 50% FBS and subjected to imaging analysis. Viruses were quantified by p24 ELISA.

### Viral RNA labeling and viral protein staining in cells

TZM-bl or TZM-rhTRIM5α cells were plated in 8-well chamber slides and virus was added at 4°C at equal multiplicities of infection or p24 levels for 30 minutes to synchronize infections. OMK cells were plated in chamber slides in the presence or absence of 10 μM CsA (Bedford Laboratories, Bedford, OH). Cells were transferred to 37°C thereafter for 15 or 30 minutes. Cells were washed twice with fresh medium and incubated at 37°C for up to 360 minutes. Cells were fixed in 2% paraformaldehyde (PFA) for 15 minutes, washed with PBS twice, and permeabilized with 0.05% Triton X in PBS for 15 minutes. The cells were stained with the Click-iT RNA Imaging Kit with Alexa Fluor 488 or Alexa Fluor 594 (Invitrogen) as per the manufacturer’s instructions. Staining with anti-CA monoclonal antibody (AG3.0), obtained through the NIH AIDS Research and Reference Reagent Program from Dr. Jonathan Allan [45], and anti-NC monoclonal antibodies, a kind gift from Dr. Robert Gorelick, was performed, followed by staining with anti-mouse IgG-Cy5. Imaging was performed using an Olympus Fluoview 1000–1 confocal microscope. RNA and viral protein puncta were enumerated by Imaris software.

### Viral RNA labeling in viral particles

EU-labeled HIV particles were treated with cell extraction buffer (100 mM sodium chloride, 10 mM Tris pH 7.5, 1 mM EDTA, 0.5% Triton X-100, 10% glycerol, 1 μg/ml pepstatin, 1 μg/ml aprotinin, 1 μg/ml leupeptin and 1 mM sodium peroxyvanadate) alone or cell lysates of 293T cells or 293T-rhTRIM5α cells (2 x10^7^ cells/ml of cell extraction buffer) for 30 minutes in the presence or absence of RNase A at room temperature. HIV-1 particles were fixed with 2% paraformaldehyde in 8-well chambered #1.0 borosilicate coverglass (Lab-Tek, Scotts Valley, CA) coated with Cell-Tak (BD Biosciences, San Jose, CA). The wells were treated with PBS containing 0.05% Triton-X-100 (Fisher Scientific, Pittsburgh, PA) for 15 minutes and washed with PBS once. vRNA was stained by the Click-iT RNA Imaging Kit per the manufacturer’s instructions and washed 5 times in PBS before mounting. Imaging was performed using an Olympus Fluoview 1000–1 Confocal Microscope and staining was enumerated using Imaris.

### qPCR for viral DNA

Viruses were treated with DNase I (60 units/ml) for 30 minutes at 37°C. To control for any contaminating plasmid carry-over, the plasmid pEGFP-C1 (Clontech, Mountain View, CA), which does not encode any retroviral sequences, was added to the virus prior to infection (6 × 10^10^ copies). TZM-bl cells were incubated with equal amounts of virus particles (5 ng CA per well) for 30 minutes at 4°C. Controls for each virus infected in the presence of 150 nM of efavirenz was included. After incubation, virus was removed and fresh medium was added. Cells were switched to 37°C for further incubation of 15 min. The plates were washed and incubated for different lengths of time. Cells were lysed with cell extraction buffer, mixed with phenol, and stored on ice. Cellular DNA was purified by phenol-chloroform extraction and ethanol precipitation. vDNA quantification by real time PCR could be affected by variations from multiple steps, including adding virus, removing virus, washing after infection, cell lysing, DNA purification and PCR sample loading. To minimize the variation in vDNA quantification, we used two primer sets in each PCR reaction to detect both reverse transcripts and internal pEGFP-C1 plasmid. The HIV-1 primers and probes used were previously described, using FAM/TAMRA [6]. The eGFP primers used were 5’-tacgtccaggagcgcaccat-3’ (forward) and 5’-cagctcgatgcggttcacca-3’ (reverse) and the probe was 5’-HEX-acgacggcaactacaagacc-IBFQ-3’. We used a single plasmid, pHIV-eGFP [46] (a kind gift from P. Bieniasz) with both HIV and GFP control sequences as the standard. After qPCR, reverse transcript levels were normalized by internal control and any background in the presence of efavirenz was subtracted, typically 2-4% of the levels measured in the absence of drug.

### Statistics

Paired two-sided student’s t tests were performed to compare puncta counts for different viral RNA or proteins, viruses, or treatment conditions at individual time points using Prism (GraphPad Software, La Jolla, CA), with the exception of comparisons of reverse transcript results which used an unpaired student’s t test of the duplicates.

## Competing interests

The authors declare they have no competing interests.

## Authors’ contributions

Conceived and designed experiments: HX, JL, CA, SW, NSC, ZA. Performed the experiments: HX, TF, GG, KH, NR, CSD. Analyzed the data: HX, CA, NSC, ZA. Wrote the paper: ZA. All authors read and approved the final manuscript.

## Supplementary Material

Additional file 1: Table S1 EU detection in MS2-GFP + virions. **Figure S1.** Toxicity of EU in 293 T cells. (A) Cells were incubated in the presence of cell culture medium containing 0.5 mM or 1 mM EU for 0, 1, or 3 h. (B) Cells were incubated in cell culture medium containing 0, 0.25, 0.5, or 1 mM EU overnight (approximately 16 h). Cell viability was determined by the XTT cell viability assay (Roche). Error bars represent standard deviations between duplicate wells. **Figure S2.** MS2-GFP binding is specific for virus with genomes containing MS2-binding sites. EU staining (red) of HIV-1 particles without (left) or with (right) MS2-binding sites produced in cells expressing MS2-GFP (green). **Figure S3.** Virus treated with cell lysate containing rhTRIM5α is sensitive to RNA degradation. EU+ puncta per field were counted for labeled virus particles treated with 293T cell extract or 293T cell extract expressing rhTRIM5α in the presence of 0, 1, 10, or 100 μg/ml RNase A. Results are representative of 2 independent experiments. Data represent the mean ± SEM of 4 fields. Significant p values (p < 0.05) are listed above each set of bars as determined by student’s t test. NS denotes p values that are not significant (p > 0.05). **Figure S4.** EU staining diffuses over time. EU staining of WT HIV-1 in TZM-bl cells at (A) 15 minutes or (B) 90 minutes post infection. Arrows denote EU staining. **Figure S5.** Example of RNA puncta and cell counts per field prior to normalization. (A) EU + puncta and (B) cells were counted in 4 fields for OMK cells treated with medium or CsA and infected with WT HIV-1. Data represent the mean ± SEM of 4 fields. Asterisks denote statistically significant p values (p < 0.05) between CsA or medium at each time point by student’s t test. Click here for file

## References

[B1] Arhel N Genovesio A Kim KA Miko S Perret E Olivo-Marin JC Shorte S Charneau P Quantitative four-dimensional tracking of cytoplasmic and nuclear HIV-1 complexes Nat Methods 2006 3 817 824 10.1038/nmeth928 16990814

[B2] McDonald D Vodicka MA Lucero G Svitkina TM Borisy GG Emerman M Hope TJ Visualization of the intracellular behavior of HIV in living cells J Cell Biol 2002 159 441 452 10.1083/jcb.200203150 12417576 PMC2173076

[B3] Levin A Loyter A Bukrinsky M Strategies to inhibit viral protein nuclear import: HIV-1 as a target Biochim Biophys Acta 1813 2011 1646 1653 10.1016/j.bbamcr.2010.07.010 PMC2994963 20719241

[B4] Forshey BM von Schwedler U Sundquist WI Aiken C Formation of a human immunodeficiency virus type 1 core of optimal stability is crucial for viral replication J Virol 2002 76 5667 5677 10.1128/JVI.76.11.5667-5677.2002 11991995 PMC137032

[B5] Dismuke DJ Aiken C Evidence for a functional link between uncoating of the human immunodeficiency virus type 1 core and nuclear import of the viral preintegration complex J Virol 2006 80 3712 3720 10.1128/JVI.80.8.3712-3720.2006 16571788 PMC1440469

[B6] Lee K Ambrose Z Martin TD Oztop I Mulky A Julias JG Vandegraaff N Baumann JG Wang R Yuen W Flexible use of nuclear import pathways by HIV-1 Cell Host Microbe 2010 7 221 233 10.1016/j.chom.2010.02.007 20227665 PMC2841689

[B7] Yamashita M Emerman M Capsid is a dominant determinant of retrovirus infectivity in nondividing cells J Virol 2004 78 5670 5678 10.1128/JVI.78.11.5670-5678.2004 15140964 PMC415837

[B8] Yamashita M Perez O Hope TJ Emerman M Evidence for direct involvement of the capsid protein in HIV infection of nondividing cells PLoS Pathog 2007 3 1502 1510 17967060 10.1371/journal.ppat.0030156 PMC2042020

[B9] Stremlau M Owens CM Perron MJ Kiessling M Autissier P Sodroski J The cytoplasmic body component TRIM5alpha restricts HIV-1 infection in Old World monkeys Nature 2004 427 848 853 10.1038/nature02343 14985764

[B10] Wu X Anderson JL Campbell EM Joseph AM Hope TJ Proteasome inhibitors uncouple rhesus TRIM5alpha restriction of HIV-1 reverse transcription and infection Proc Natl Acad Sci U S A 2006 103 7465 7470 10.1073/pnas.0510483103 16648264 PMC1464362

[B11] Warrilow D Stenzel D Harrich D Isolated HIV-1 core is active for reverse transcription Retrovirology 2007 4 77 10.1186/1742-4690-4-77 17956635 PMC2169257

[B12] Warrilow D Warren K Harrich D Strand transfer and elongation of HIV-1 reverse transcription is facilitated by cell factors in vitro PLoS One 2010 5 e13229 10.1371/journal.pone.0013229 20949087 PMC2950853

[B13] Hulme AE Perez O Hope TJ Complementary assays reveal a relationship between HIV-1 uncoating and reverse transcription Proc Natl Acad Sci U S A 2011 108 9975 9980 10.1073/pnas.1014522108 21628558 PMC3116424

[B14] Albanese A Arosio D Terreni M Cereseto A HIV-1 pre-integration complexes selectively target decondensed chromatin in the nuclear periphery PLoS One 2008 3 e2413 10.1371/journal.pone.0002413 18545681 PMC2398779

[B15] Arhel NJ Souquere-Besse S Munier S Souque P Guadagnini S Rutherford S Prevost MC Allen TD Charneau P HIV-1 DNA Flap formation promotes uncoating of the pre-integration complex at the nuclear pore EMBO J 2007 26 3025 3037 10.1038/sj.emboj.7601740 17557080 PMC1894778

[B16] Campbell EM Perez O Anderson JL Hope TJ Visualization of a proteasome-independent intermediate during restriction of HIV-1 by rhesus TRIM5alpha J Cell Biol 2008 180 549 561 10.1083/jcb.200706154 18250195 PMC2234241

[B17] Jao CY Salic A Exploring RNA transcription and turnover in vivo by using click chemistry Proc Natl Acad Sci U S A 2008 105 15779 15784 10.1073/pnas.0808480105 18840688 PMC2572917

[B18] Onafuwa-Nuga AA Telesnitsky A King SR 7SL RNA, but not the 54-kd signal recognition particle protein, is an abundant component of both infectious HIV-1 and minimal virus-like particles RNA (New York, NY) 2006 12 542 546 10.1261/rna.2306306 PMC1421090 16489186

[B19] Rulli SJ Jr Hibbert CS Mirro J Pederson T Biswal S Rein A Selective and nonselective packaging of cellular RNAs in retrovirus particles J Virol 2007 81 6623 6631 10.1128/JVI.02833-06 17392359 PMC1900105

[B20] Svarovskaia ES Xu H Mbisa JL Barr R Gorelick RJ Ono A Freed EO Hu WS Pathak VK Human apolipoprotein B mRNA-editing enzyme-catalytic polypeptide-like 3G (APOBEC3G) is incorporated into HIV-1 virions through interactions with viral and nonviral RNAs J Biol Chem 2004 279 35822 35828 10.1074/jbc.M405761200 15210704

[B21] Black LR Aiken C TRIM5alpha disrupts the structure of assembled HIV-1 capsid complexes in vitro J Virol 2010 84 6564 6569 10.1128/JVI.00210-10 20410272 PMC2903270

[B22] Langelier CR Sandrin V Eckert DM Christensen DE Chandrasekaran V Alam SL Aiken C Olsen JC Kar AK Sodroski JG Sundquist WI Biochemical characterization of a recombinant TRIM5alpha protein that restricts human immunodeficiency virus type 1 replication J Virol 2008 82 11682 11694 10.1128/JVI.01562-08 18799573 PMC2583683

[B23] Zhao G Ke D Vu T Ahn J Shah VB Yang R Aiken C Charlton LM Gronenborn AM Zhang P Rhesus TRIM5alpha disrupts the HIV-1 capsid at the inter-hexamer interfaces PLoS Pathog 2011 7 e1002009 10.1371/journal.ppat.1002009 21455494 PMC3063768

[B24] Dahlberg JE Quantitative electron microscopic analysis of the penetration of VSV into L cells Virology 1974 58 250 262 10.1016/0042-6822(74)90159-7 4362549

[B25] Fan DP Sefton BM The entry into host cells of Sindbis virus, vesicular stomatitis virus and Sendai virus Cell 1978 15 985 992 10.1016/0092-8674(78)90282-9 215317

[B26] Campbell EM Perez O Melar M Hope TJ Labeling HIV-1 virions with two fluorescent proteins allows identification of virions that have productively entered the target cell Virology 2007 360 286 293 10.1016/j.virol.2006.10.025 17123568 PMC1885464

[B27] Levin JG Guo J Rouzina I Musier-Forsyth K Nucleic acid chaperone activity of HIV-1 nucleocapsid protein: critical role in reverse transcription and molecular mechanism Prog Nucleic Acid Res Mol Biol 2005 80 217 286 16164976 10.1016/S0079-6603(05)80006-6

[B28] Ganser-Pornillos BK Chandrasekaran V Pornillos O Sodroski JG Sundquist WI Yeager M Hexagonal assembly of a restricting TRIM5alpha protein Proc Natl Acad Sci U S A 2011 108 534 539 10.1073/pnas.1013426108 21187419 PMC3021009

[B29] Nisole S Lynch C Stoye JP Yap MW A Trim5-cyclophilin A fusion protein found in owl monkey kidney cells can restrict HIV-1 Proc Natl Acad Sci U S A 2004 101 13324 13328 10.1073/pnas.0404640101 15326303 PMC516566

[B30] Sayah DM Sokolskaja E Berthoux L Luban J Cyclophilin A retrotransposition into TRIM5 explains owl monkey resistance to HIV-1 Nature 2004 430 569 573 10.1038/nature02777 15243629

[B31] Dudding LR Nkabinde NC Mizrahi V Analysis of the RNA- and DNA-dependent DNA polymerase activities of point mutants of HIV-1 reverse transcriptase lacking ribonuclease H activity Biochemistry 1991 30 10498 10506 10.1021/bi00107a019 1718422

[B32] Shi J Zhou J Shah VB Aiken C Whitby K Small-molecule inhibition of human immunodeficiency virus type 1 infection by virus capsid destabilization J Virol 2011 85 542 549 10.1128/JVI.01406-10 20962083 PMC3014163

[B33] Jun S Ke D Debiec K Zhao G Meng X Ambrose Z Gibson GA Watkins SC Zhang P Direct visualization of HIV-1 infection using correlative live-cell microscopy and cryo-electron microscopy Structure 2011 19 1573 1581 10.1016/j.str.2011.09.006 22078557 PMC3217200

[B34] Yang R Shi J Byeon IJ Ahn J Sheehan JH Meiler J Gronenborn AM Aiken C Second-site suppressors of HIV-1 capsid mutations: restoration of intracellular activities without correction of intrinsic capsid stability defects Retrovirology 2012 9 30 10.1186/1742-4690-9-30 22515365 PMC3351742

[B35] Blair WS Pickford C Irving SL Brown DG Anderson M Bazin R Cao J Ciaramella G Isaacson J Jackson L HIV capsid is a tractable target for small molecule therapeutic intervention PLoS Pathog 2010 6 e1001220 10.1371/journal.ppat.1001220 21170360 PMC3000358

[B36] von Schwedler UK Stray KM Garrus JE Sundquist WI Functional surfaces of the human immunodeficiency virus type 1 capsid protein J Virol 2003 77 5439 5450 10.1128/JVI.77.9.5439-5450.2003 12692245 PMC153941

[B37] Yap MW Dodding MP Stoye JP Trim-cyclophilin A fusion proteins can restrict human immunodeficiency virus type 1 infection at two distinct phases in the viral life cycle J Virol 2006 80 4061 4067 10.1128/JVI.80.8.4061-4067.2006 16571822 PMC1440439

[B38] Mirambeau G Lyonnais S Gorelick RJ Features, processing states, and heterologous protein interactions in the modulation of the retroviral nucleocapsid protein function RNA Biol 2010 7 724 734 10.4161/rna.7.6.13777 21045549 PMC3073331

[B39] Kutluay SB Perez-Caballero D Bieniasz PD Fates of retroviral core components during unrestricted and TRIM5-restricted infection PLoS Pathog 2013 9 e1003214 10.1371/journal.ppat.1003214 23505372 PMC3591316

[B40] Shah VB Shi J Hout DR Oztop I Krishnan L Ahn J Shotwell MS Engelman A Aiken C The host proteins transportin SR2/TNPO3 and cyclophilin A exert opposing effects on HIV-1 uncoating J Virol 2013 87 422 432 10.1128/JVI.07177-11 23097435 PMC3536424

[B41] Wu X Liu H Xiao H Conway JA Hunter E Kappes JC Functional RT and IN incorporated into HIV-1 particles independently of the Gag/Pol precursor protein EMBO J 1997 16 5113 5122 10.1093/emboj/16.16.5113 9305652 PMC1170145

[B42] Cecilia D KewalRamani VN O'Leary J Volsky B Nyambi P Burda S Xu S Littman DR Zolla-Pazner S Neutralization profiles of primary human immunodeficiency virus type 1 isolates in the context of coreceptor usage J Virol 1998 72 6988 6996 9696790 10.1128/jvi.72.9.6988-6996.1998 PMC109918

[B43] Bartz SR Vodicka MA Production of high-titer human immunodeficiency virus type 1 pseudotyped with vesicular stomatitis virus glycoprotein Methods (San Diego, Calif) 1997 12 337 342 10.1006/meth.1997.0487 9245614

[B44] Xu H Svarovskaia ES Barr R Zhang Y Khan MA Strebel K Pathak VK A single amino acid substitution in human APOBEC3G antiretroviral enzyme confers resistance to HIV-1 virion infectivity factor-induced depletion Proc Natl Acad Sci U S A 2004 101 5652 5657 10.1073/pnas.0400830101 15054139 PMC397464

[B45] Simm M Shahabuddin M Chao W Allan JS Volsky DJ Aberrant Gag protein composition of a human immunodeficiency virus type 1 vif mutant produced in primary lymphocytes J Virol 1995 69 4582 4586 7769728 10.1128/jvi.69.7.4582-4586.1995 PMC189210

[B46] Zhang YJ Hatziioannou T Zang T Braaten D Luban J Goff SP Bieniasz PD Envelope-dependent, cyclophilin-independent effects of glycosaminoglycans on human immunodeficiency virus type 1 attachment and infection J Virol 2002 76 6332 6343 10.1128/JVI.76.12.6332-6343.2002 12021366 PMC136233

