# Supplementary material for: Evidence for biphasic uncoating during HIV-1 infection from a novel imaging assay
Source: Retrovirology. 2013 Jul 9;10:70. doi: 10.1186/1742-4690-10-70 (PMC3716918; doi:10.1186/1742-4690-10-70)
Supplement: Additional file 1: Table S1 — EU detection in MS2-GFP + virions. Figure S1. Toxicity of EU in 293 T cells. (A) Cells were incubated in the presence of cell culture medium containing 0.5 mM or 1 mM EU for 0, 1, or 3 h. (B) Cells were incubated in cell culture medium containing 0, 0.25, 0.5, or 1 mM EU overnight (approximately 16 h). Cell viability was determined by the XTT cell viability assay (Roche). Error bars represent standard deviations between duplicate wells. Figure S2. MS2-GFP binding is specific for virus with genomes containing MS2-binding sites. EU staining (red) of HIV-1 particles without (left) or with (right) MS2-binding sites produced in cells expressing MS2-GFP (green). Figure S3. Virus treated with cell lysate containing rhTRIM5α is sensitive to RNA degradation. EU+ puncta per field were counted for labeled virus particles treated with 293T cell extract or 293T cell extract expressing rhTRIM5α in the presence of 0, 1, 10, or 100 μg/ml RNase A. Results are representative of 2 independent experiments. Data represent the mean ± SEM of 4 fields. Significant p values (p < 0.05) are listed above each set of bars as determined by student’s t test. NS denotes p values that are not significant (p > 0.05). Figure S4. EU staining diffuses over time. EU staining of WT HIV-1 in TZM-bl cells at (A) 15 minutes or (B) 90 minutes post infection. Arrows denote EU staining. Figure S5. Example of RNA puncta and cell counts per field prior to normalization. (A) EU + puncta and (B) cells were counted in 4 fields for OMK cells treated with medium or CsA and infected with WT HIV-1. Data represent the mean ± SEM of 4 fields. Asterisks denote statistically significant p values (p < 0.05) between CsA or medium at each time point by student’s t test. [file 1742-4690-10-70-S1.ppt]

## Slide 1
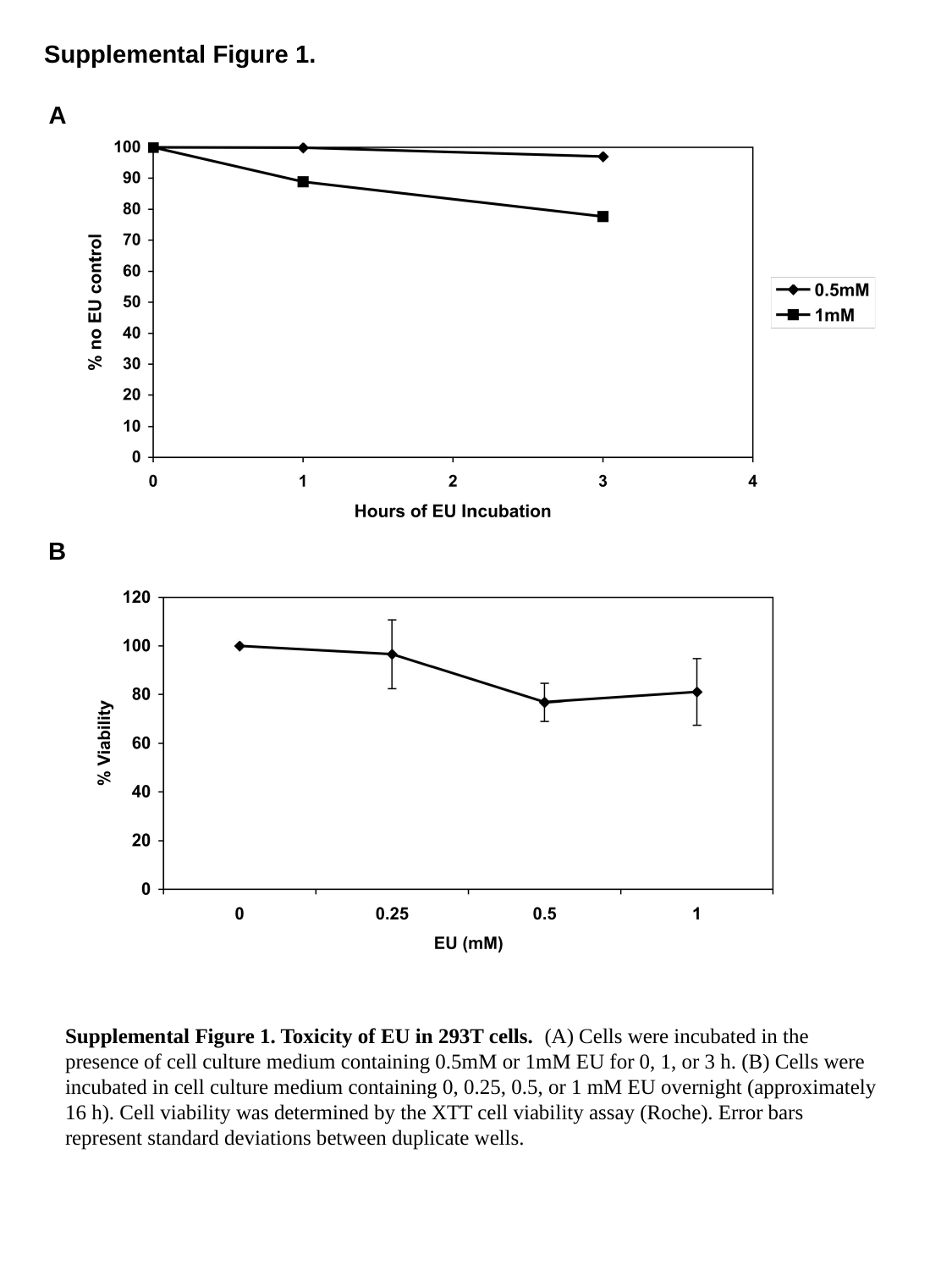

Supplemental Figure 1.
A
B
Supplemental Figure 1. Toxicity of EU in 293T cells. (A) Cells were incubated in the presence of cell culture medium containing 0.5mM or 1mM EU for 0, 1, or 3 h. (B) Cells were incubated in cell culture medium containing 0, 0.25, 0.5, or 1 mM EU overnight (approximately 16 h). Cell viability was determined by the XTT cell viability assay (Roche). Error bars represent standard deviations between duplicate wells.

## Slide 2
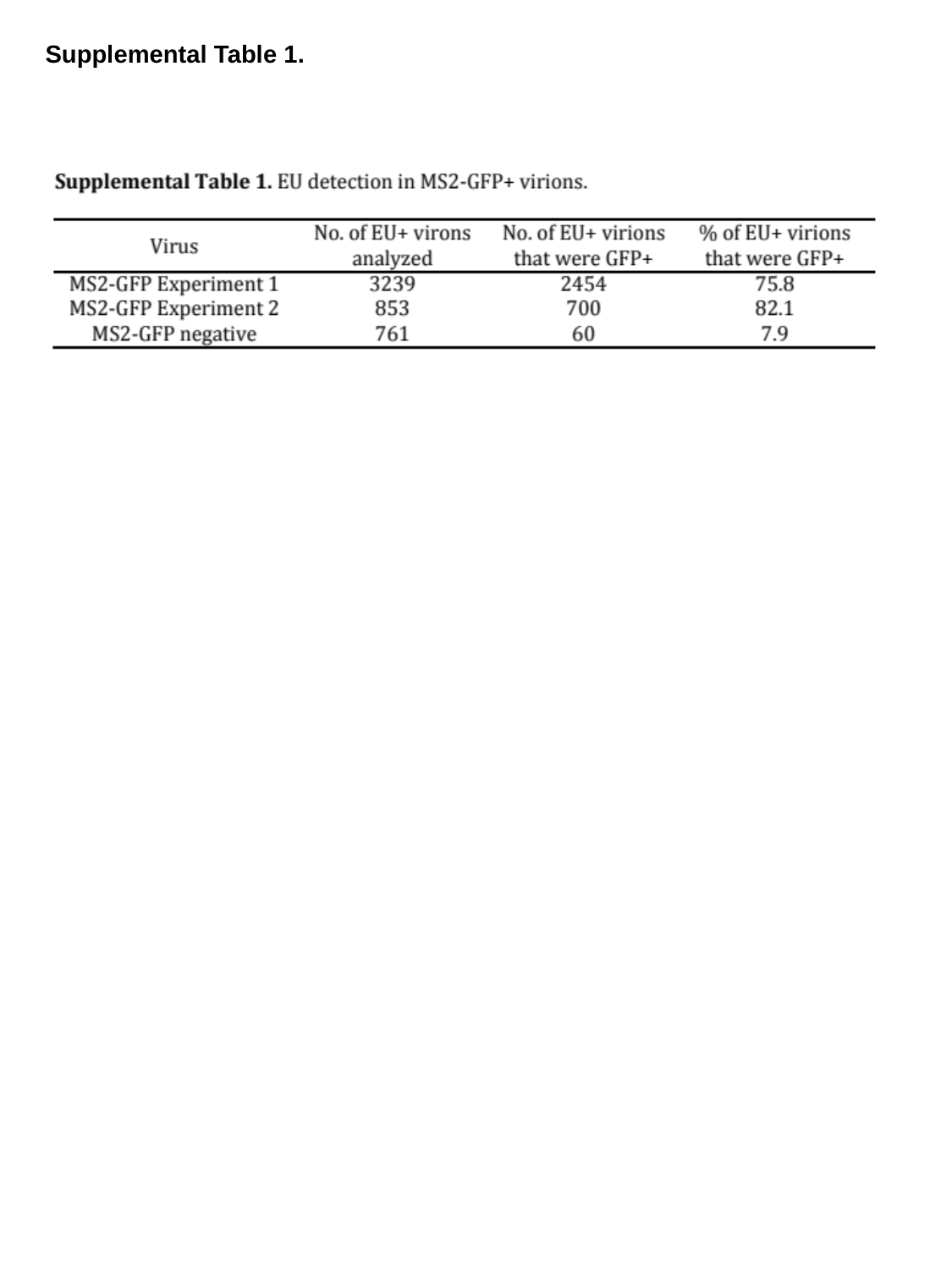

Supplemental Table 1.

## Slide 3
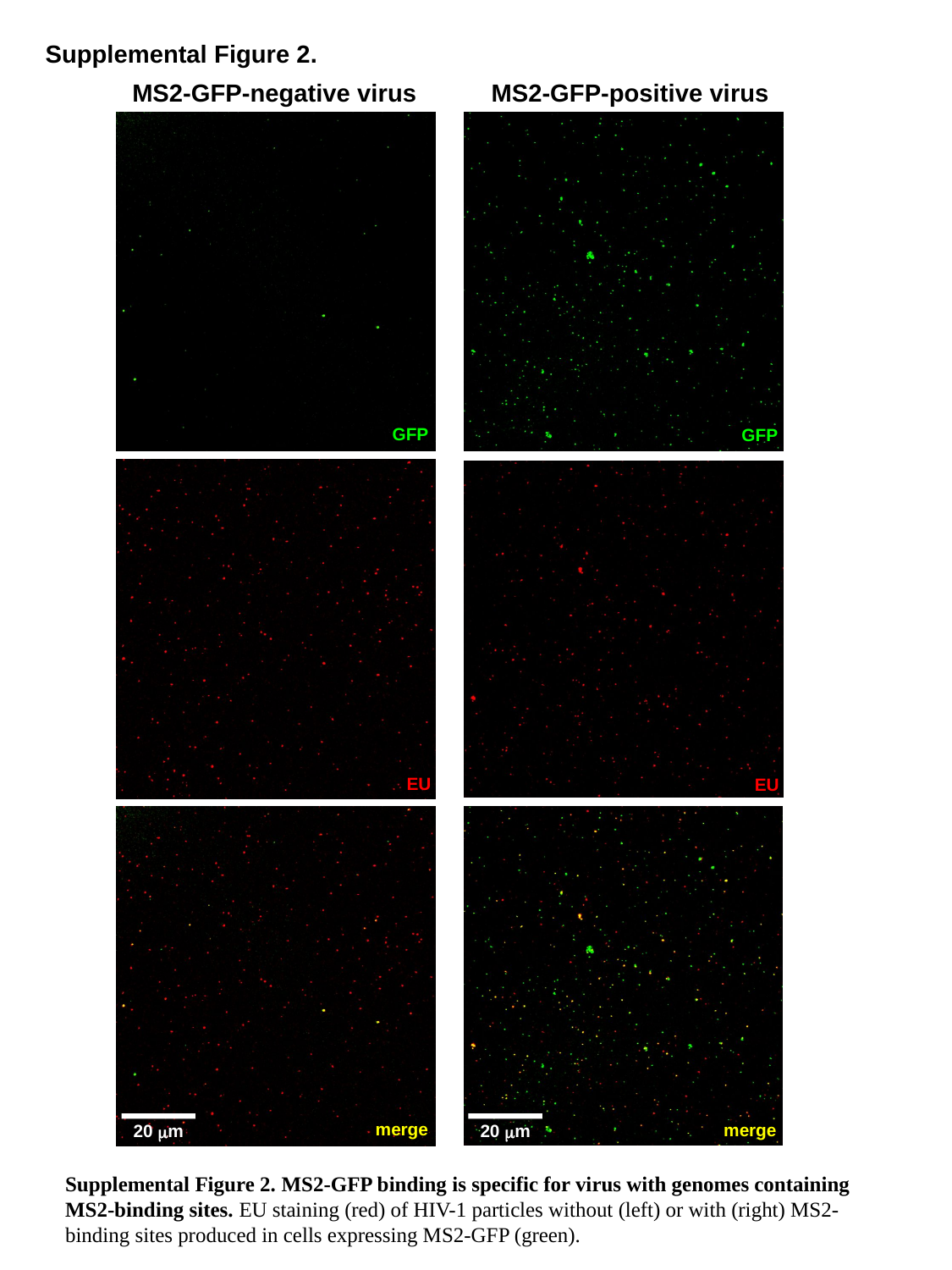

Supplemental Figure 2.
MS2-GFP-negative virus
MS2-GFP-positive virus
GFP
GFP
EU
EU
merge
merge
20 μm
20 μm
Supplemental Figure 2. MS2-GFP binding is specific for virus with genomes containing MS2-binding sites. EU staining (red) of HIV-1 particles without (left) or with (right) MS2-binding sites produced in cells expressing MS2-GFP (green).

## Slide 4
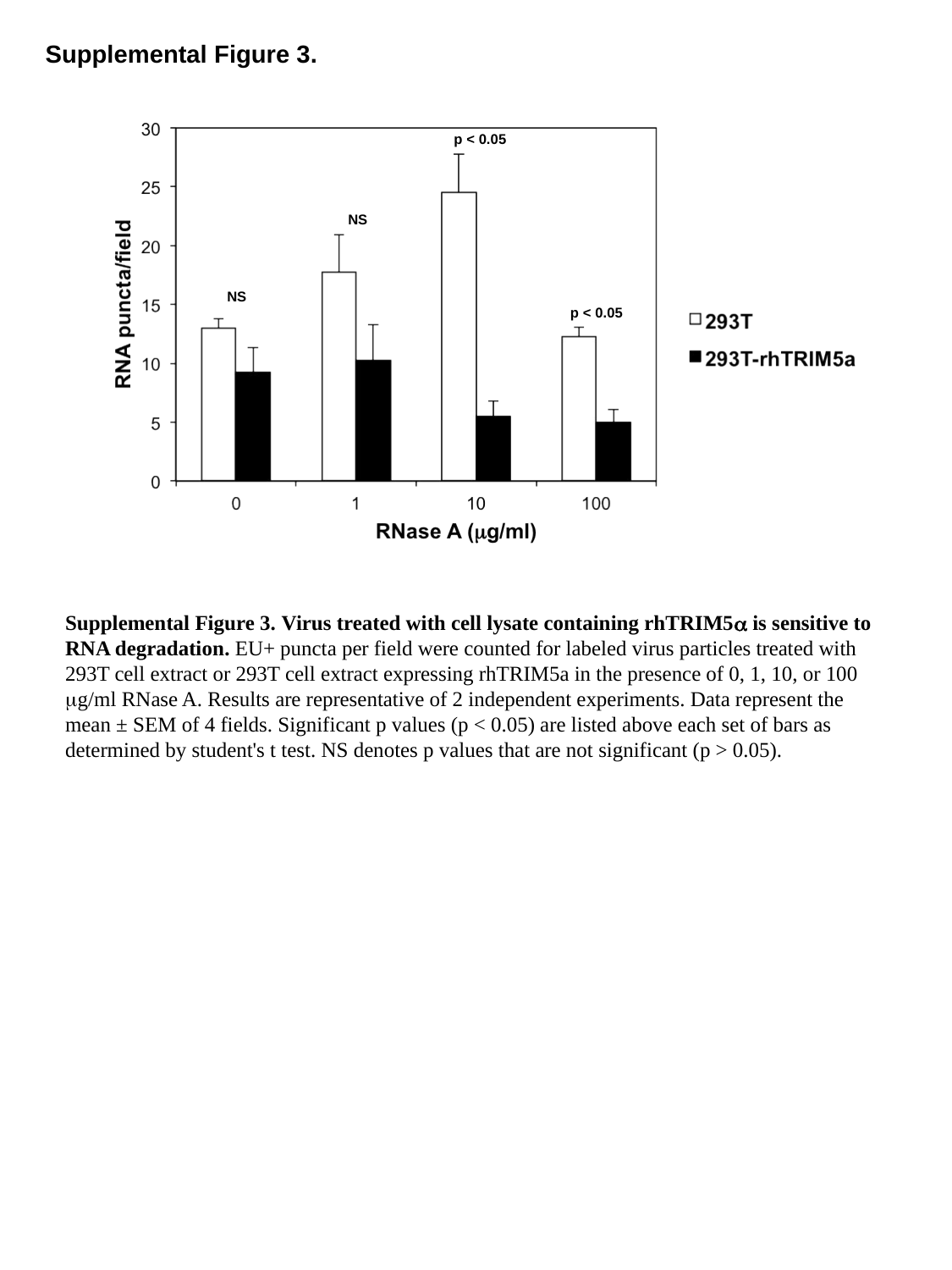

Supplemental Figure 3.
p < 0.05
NS
NS
p < 0.05
Supplemental Figure 3. Virus treated with cell lysate containing rhTRIM5α is sensitive to RNA degradation. EU+ puncta per field were counted for labeled virus particles treated with 293T cell extract or 293T cell extract expressing rhTRIM5a in the presence of 0, 1, 10, or 100 μg/ml RNase A. Results are representative of 2 independent experiments. Data represent the mean ± SEM of 4 fields. Significant p values (p < 0.05) are listed above each set of bars as determined by student's t test. NS denotes p values that are not significant (p > 0.05).

## Slide 5
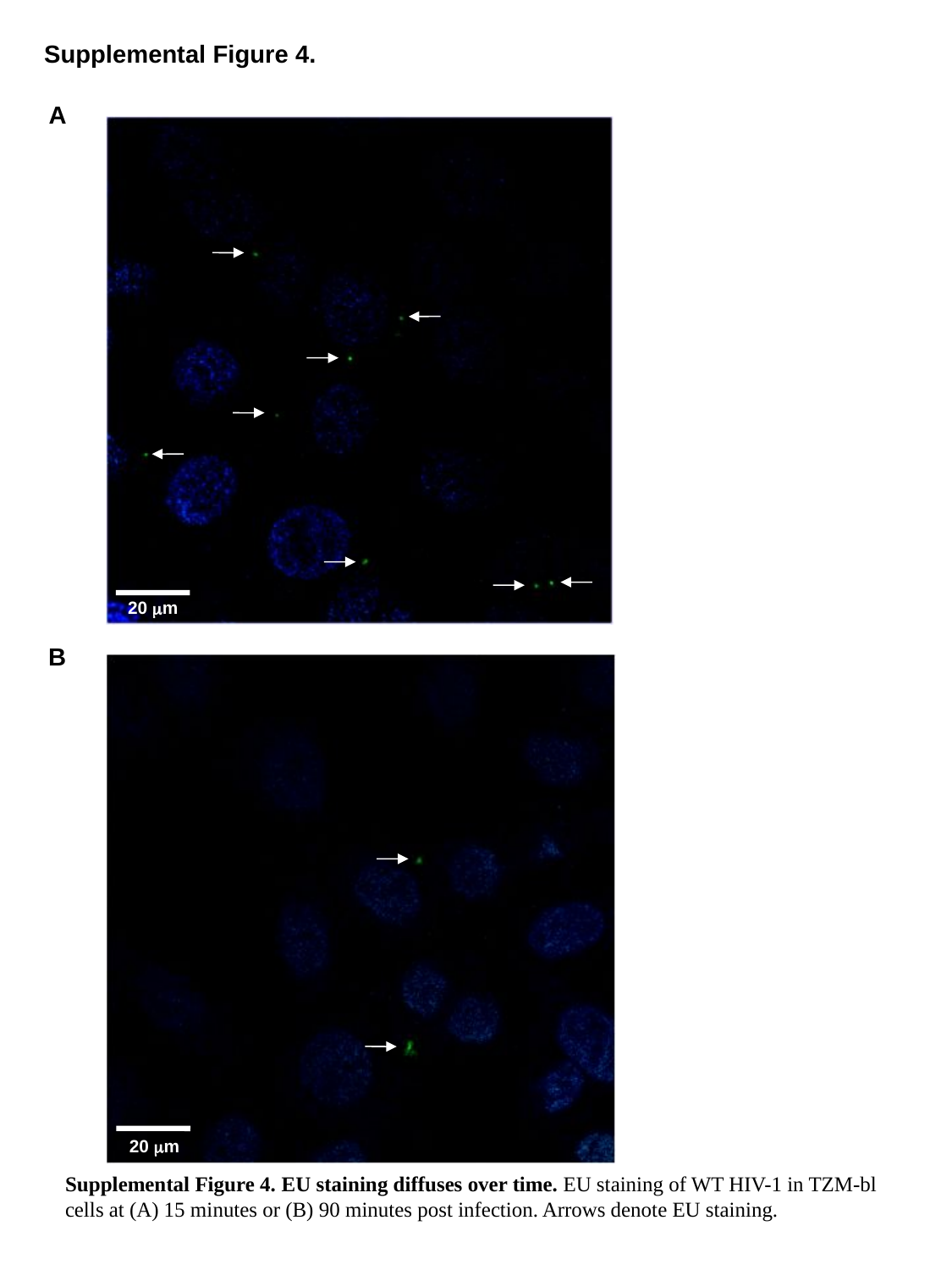

Supplemental Figure 4.
A
20 μm
B
20 μm
Supplemental Figure 4. EU staining diffuses over time. EU staining of WT HIV-1 in TZM-bl cells at (A) 15 minutes or (B) 90 minutes post infection. Arrows denote EU staining.

## Slide 6
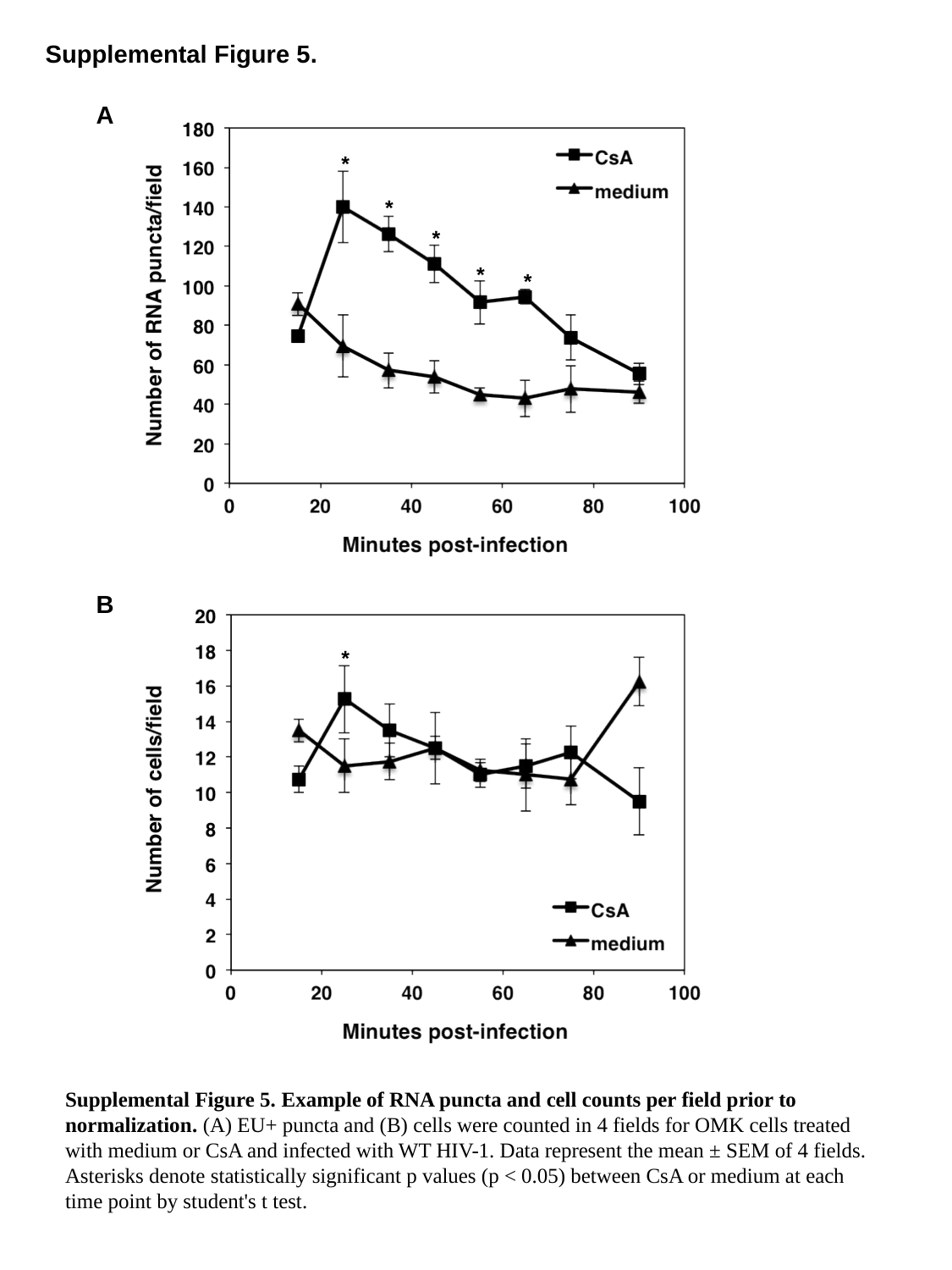

Supplemental Figure 5.
A
*
*
*
*
*
B
*
Supplemental Figure 5. Example of RNA puncta and cell counts per field prior to normalization. (A) EU+ puncta and (B) cells were counted in 4 fields for OMK cells treated with medium or CsA and infected with WT HIV-1. Data represent the mean ± SEM of 4 fields. Asterisks denote statistically significant p values (p < 0.05) between CsA or medium at each time point by student's t test.
